# SNORA71A Promotes Colorectal Cancer Cell Proliferation, Migration, and Invasion

**DOI:** 10.1155/2020/8284576

**Published:** 2020-10-05

**Authors:** Zhengxiang Zhang, Yunxiang Tao, Qingling Hua, Juan Cai, Xiaobing Ye, Hao Li

**Affiliations:** ^1^Department of Oncology, Yijishan Hospital of Wannan Medical College, Wuhu, Anhui, China; ^2^Department of Dermatology, Yijishan Hospital of Wannan Medical College, Wuhu, Anhui, China; ^3^College of Food Engineering, Jilin Engineering Normal University, Changchun, China

## Abstract

Small nucleolar RNAs (snoRNAs) play a crucial role during colorectal cancer (CRC) development. The study of SNORA71A is few, and its role in CRC is unknown. This study focused on screening abnormal snoRNAs in CRC and exploring the role of key snoRNA in CRC. The expression pattern of snoRNAs in 3 CRC and 3 normal colon tissues was detected via small RNA sequencing. The six candidate snoRNAs were identified by quantitative PCR (qPCR). Subsequently, the expression level of SNORA71A was further verified through the Cancer Genome Atlas (TCGA) data analysis and qPCR. The CCK8 and transwell assays were used to detect the functional role of SNORA71A in CRC cells. The integrated analysis of snoRNA expression profile indicated that a total 107 snoRNAs were significantly differentially expressed (DE) in CRC tissues compared with normal tissues, including 45 upregulated and 62 downregulated snoRNAs. Bioinformatics analysis revealed that the DE snoRNAs were mainly implicated in “detection of chemical stimulus involved in sensory perception of smell” and “sensory perception of smell” in the biological process. The DE snoRNAs were preferentially enriched in “olfactory transduction” and “glycosphingolipid biosynthesis-ganglio series pathway.” The expression of SNORA71A was upregulated in CRC tissues and cells. SNORA71A expression showed statistically significant correlations with TNM stage (*P* = 0.0196) and lymph node metastasis (*P* = 0.0189) and can serve as biomarkers for CRC. Importantly, SNORA71A significantly facilitated the CRC cell proliferation, migration, and invasion. Our findings indicate that SNORA71A screened by sequencing acted as an oncogene and promoted proliferation, migration, and invasion ability of CRC cells.

## 1. Introduction

Colorectal cancer (CRC) is one of the most common types of human cancers with approximately 1800 thousands of new cases each year and >860,000 deaths worldwide [1]. The CRC has increased morbidity and mortality rapidly in recent years, with high mortality rates (2007-2016) of 35%, 45%, and 47.8% in the USA, Europe, and worldwide, respectively [1, 2] (European Cancer Information [3]). In the past decades, CRC patients are well treated with the progress of CRC diagnostic technologies and therapeutic strategies [4]. However, high recurrent disease rates and low survival rates of CRC remaine a severe problem that affected the life quality of CRC patients. Therefore, there is still an urgent need to develop novel effective treatment aimed at CRC patients, especially patients with metastatic colorectal cancer [5, 6].

The epigenetic changes and altered expression of noncoding RNAs (ncRNAs) have important pathophysiological roles in the initiation and progression of CRC and ultimately lead to disease metastasis [7, 8]. An extensive body of research show that ncRNAs regulate epithelial proliferation checkpoints, epithelial-mesenchymal metastasis, and inflammatory gene expression in CRC [9]. For instance, lncRNA GAS5 regulates the upstream P53 involving CRC cell cycle arrest [10]. The decreased miR-34a can enhance TGF*β* signaling and tumor CRC cell invasion and promote EMT [11]. lncRNA NEAT1 enhances IL-6 expression through activating the JNK1/2 and ERK1/2 signaling cascade pathways in CRC [12]. However, it is not known whether small nucleolar RNAs (snoRNAs) have also been implicated in the initiation and progress of CRC.

SnoRNAs encoded in the intron of the host gene are a class of about 60 to 300 nucleotides in length and have functions of posttranscriptional modification in ribosomal RNAs and some spliceosomal RNAs [13, 14]. The emerging studies have demonstrated that abnormal snoRNAs may play important roles in human malignancies, including CRC. For instance, SNORA42 is overexpressed in colorectal cancer and promotes CRC cell proliferation, invasion, and migration [15]. SNHG6 also encodes snoRNAs and is upregulated in CRC [16]. SNHG1 inhibits the expression of miR-154-5p and promotes CRC cell cycleprogression [17, 18]. However, little is known on about the functions and mechanisms of snoRNAs implicated with CRC.

In this study, using small RNA sequencing, we found that the SNORA71A was upregulated in CRC tissues and significantly differentially expressed. The SNORA71A expression was further validated by RT-qPCR and TCGA data analysis in CRC clinical samples. Furthermore, the potential targets and signaling pathway of SNORA71A were analyzed through bioinformatics. The functional effect of SNORA71A on CRC was evaluated by cell proliferation, migration, and invasion assays.

## 2. Materials and Methods

### 2.1. Patient and Clinical Sample Collection

Colon tissue samples of 3 cases of CRC patients and 3 cases of normal subjects were obtained from the Yijishan Hospital of Wannan Medical College. This study was approved by the Yijishan Hospital of Wannan Medical College. Consent forms were obtained from all patients for participating in this study.

### 2.2. RNA Isolation, Library Construction, and Small RNA Sequencing

Total RNA was isolated using TRIzol Plus RNA Purification Kit (Cat. 12183555. Thermo Fisher) according to the manufacturer's instructions and then quantified. For library preparation using TruSeq Small RNA Sample Preparation Kit (Illumina), rRNA was removed from total RNA. Next, the RNA was ligated with adaptors; reverse transcribed and unique indexes were incorporated during PCR amplification. The electrophoresis was further performed to excise the snoRNA from gel region. Finally, the small RNA libraries were sequenced on an Illumina HiSeq 2000 instrument at Ying Biotech (Shanghai, China).

### 2.3. Data Analysis

The qualities of raw sequencing reads were evaluated by Fast-QC (http://www.bioinformatics.babraham.ac.uk/projects/fastqc), and low-quality reads were further removed. The clean small RNA reads were mapped to the miRBase database (http://www.mirbase.org/); the unmapped reads of the former step were matched to the piRNA cluster (http://www.smallrnagroup.uni-mainz.de/piRNAclusterDB.html), following Genomic tRNA database (GtRNAdb, http://gtrnadb.ucsc.edu/) and tRFdb (http://genome.bioch.virginia.edu/trfdb/) in order. Finally, the unmapped reads were scheduled to Rfam (http://rfam.xfam.org/) to identify small nucleolar RNA (snoRNA) as well as small nuclear RNA (snRNA) sequences.

### 2.4. Prediction of Targeted Genes and Functional Annotation

The two algorithms (MiRanda and RNAhybrid) were applied for snoRNA target prediction, with the cutoff criteria of score ≥ 150 and energy < −20 for MiRanda and energy < −25 for RNAhybrid. The differentially expressed (DE) snoRNAs were analyzed based on the TCGA database. Then, the top100 targeted genes of snoRNAs were analyzed using GO and KEGG analysis for these target genes. The GO database (http://geneontology.org) was performed to annotate the functions of differentially expressed (DE) snoRNA target genes, containing Molecular Function (MF), biological process (BP), and cellular component (CC). The KEGG pathway database (http://www.genome.jp/Kegg/) was utilized to systematically evaluate the predominant pathway.

### 2.5. Verification of Candidate snoRNA Expression by Quantitative PCR

The expression of the five snoRNAs was verified by quantitative PCR (qPCR) between the sequencing CRC tissue samples and normal tissue samples. The total RNA was extracted from tissue samples using the TRIZOL reagent (Invitrogen, CA, USA) according to the manufacturer's protocol. The extracted RNA was performed for quality control, and the integrity of RNA was assessed via agarose gel electrophoresis. The 1 *μ*g RNA was then used for reverse transcribe with a commercial reverse transcription kit made by Thermo Bio, followed by qPCR (2 × Master Mix kit from Roche) according to the manufacturer's instructions. FastStart Universal SYBR Green Master mix was used to amplify cDNA on QuantStudio 6 Flex Real-Time PCR System (Thermo Fisher, Shanghai, China). GAPDH was used as the endogenous control. The expression levels of the relative genes were analyzed by the 2^-∆∆Ct^ method. Each experiment was performed three times. The primer sequences used are presented in Table 1.

### 2.6. Verification through TCGA Database and on Surgical Tissues by qPCR

According to 443 CRC cancer tissue and 40 normal tissue data from the TCGA database, the expressions of the screened snoRNAs in CRC tissues compared with that in normal tissues was analyzed. The expression of that was further verified by qPCR between 36 pairs of CRC tissues and normal tissues.

### 2.7. Cell Culture

The SW620, SW480, HCT116, LOVO, and HT-29 human CRC cell lines as well as NCN460 normal colonic epithelial cells were obtained from Shanghai Genechem Co. Ltd. (Shanghai, China). The CRC cell lines were cultured in L-15 medium (Gibco, Carlsbad, USA), and NCN460 cell lines were grown in RPMI 1640 (Gibco BRL). The medium were supplemented with 10% fetal bovine serum (Gibco, 10099-14) and 1% penicillin-streptomycin (Gibco) and incubated at 37°C and 5% CO_2_.

### 2.8. Interference, Overexpression, and Transfections

For the interference process, small interfering RNAs of SNORA71A (si-SNORA71A) and the negative control (NC) (GenePharma, Shanghai, China) were transiently transfected into HT-29 cell lines by using Lipofectamine 2000 (Invitrogen, Carlsbad, CA, USA), following the manufacturer's instructions, respectively. The SNORA71A was amplified, cloned into the plasmid pcDNA3.1, and transfected into SW480 cell lines. Interference or overexpression efficiency was evaluated at the mRNA level by qPCR for subsequent experiments. The siRNA sequences are listed in Table 1.

### 2.9. Cell Counting Kit-8 (CCK8) Assay

For cell proliferation assay, 2 × 10^4^ cells per well were seeded in a 96-well plate. After SW480 cell overexpression and HT-29 cell interference, the number was detected with Cell Counting Kit-8 (CCK8) (Beyotime, Shanghai, China) for 5 consecutive days. The absorbance at 450 nm was measured with Infinite M1000 instrument (Tecan Austria GmbH, Grödig, Austria).

### 2.10. Migration and Invasion Assays

For migration and invasion assay, 2 × 10^5^ cells resuspended in serum-free media were seeded into the upper chamber of the transwell, while the full media containing 10% FBS were added to the bottom chamber to present chemoattractants for migration and invasion. After SW480 cell overexpression and HT-29 cell interference, the migrated and invasion cells were fixed with methanol and stained with crystal violet (Beyotime, Shanghai, China) for cell counting at 24 h. The basic steps of cell migration and invasion assays were largely the same except that the transwell chamber was coated with Matrigel and migration chamber was not.

### 2.11. Constructed Network of SNORA71A-mRNA-Pathway

The Cytoscape 2.8.3 was used to establish the SNORA71A/mRNA/pathway interaction network.

### 2.12. Statistical Analyses

The data were analyzed using GraphPad Prism v.8.0 (GraphPad Software, San Diego, CA). The data were expressed as the mean ± standard deviation (SD). Student's *t*-test was applied for two groups. The one-way analysis of variance (ANOVA) followed by Tukey's multiple comparison test was applied for more than two groups. The difference was considered statistically significant at *P* < 0.05.

## 3. Results

### 3.1. Expression Profile Change of snoRNAs in the CRC Tissues

To explore abnormally expressed snoRNAs in CRC tissues compared to normal tissues, we conducted small RNA sequencing. A total of 133,679,270, 156,613,612, and 30,570,028 raw reads were obtained in three normal colon tissue samples (N1, N2, and N3), and 48,668,926, 44,924,294, and 25,934,552 raw reads were gained, respectively, in the three CRC tissue samples (T1, T2, and T3). After quality assessment and filtering ~35 million clean reads, each sample was utilized to analyze via FASTQC software. The GC content of clean reads ranged from 47 to 49% in the snoRNA libraries (Table 2).

**Figure 1:**
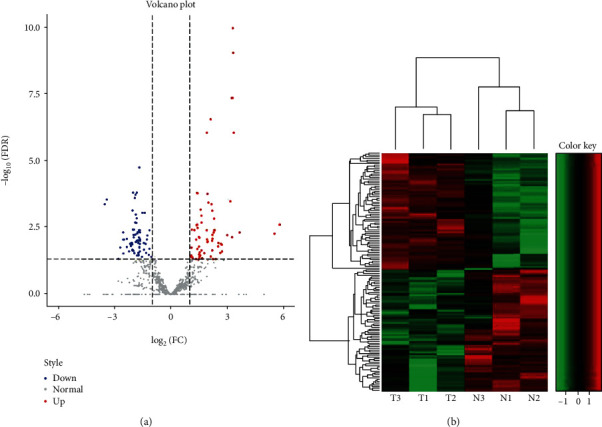
Differentially expressed snoRNAs in CRC. (a) The volcano plot shows the differentially expressed snoRNAs in CRC. Red dots and blue dots represent the upregulated and downregulated snoRNAs, respectively. The log2 (fold change) was ≥1 and FDR ≤ 0.05. (b) The heat map of the differentially expressed (DE) snoRNAs in CRC. N1, N2, and N3 represents the normal colon tissue samples; T1, T2, and T3 showed the CRC tissue samples. Green: downregulated snoRNAs; red upregulated snoRNAs.

Compared to the normal colon tissues, large perturbations of the snoRNA profile were identified from the CRC tissues via deep sequencing. we found 107 DE snoRNAs that were significantly changed, 45 DE snoRNAs were significantly upregulated, and 62 were significantly downregulated (Supplementary 1) (Figure 1(a)). By performing a hierarchical cluster analysis, the heat map exhibited similar clusters within groups and significant differences between CRC and normal samples, indicating different snoRNA-regulation patterns in the two groups (Figure 1(b)).

### 3.2. Functional Enrichment, Pathway, and the Candidate snoRNA Validation

**Figure 2:**
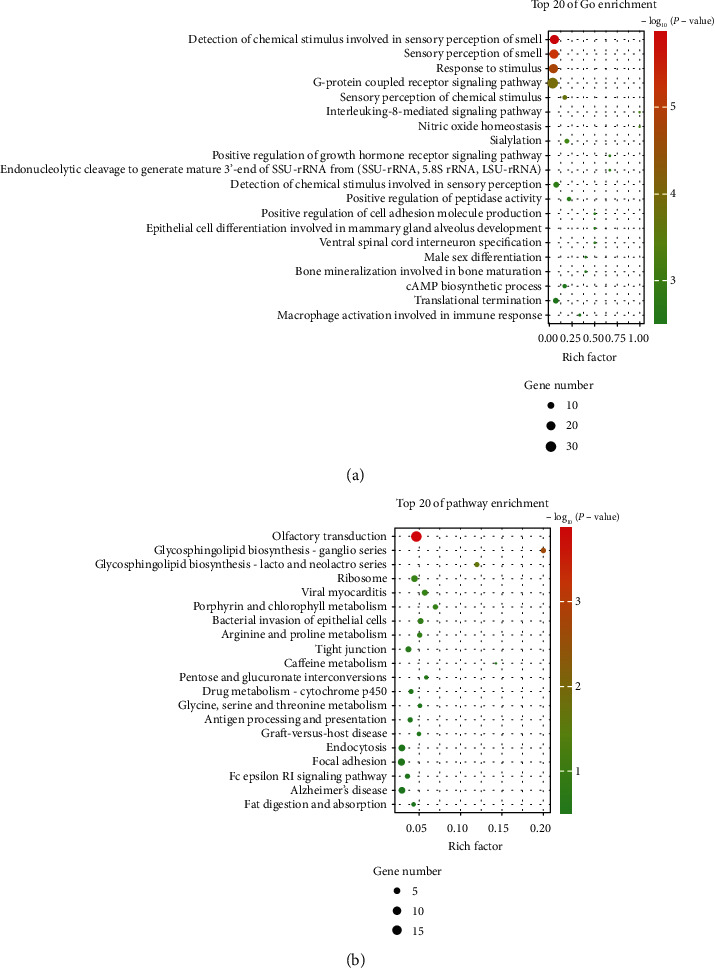
Functional enrichment analysis of snoRNA-associated target genes. (a) GO functional enrichment analysis on predicted target genes of snoRNAs. The *x*-axis shows the enrichment factor including gene numbers and -log10 (*P* value), and the *y*-axis presents the top 20 GO enrichment terms. (b) Top 20 significantly enriched KEGG pathways. The horizontal axis refers to the enrichment factor, and the vertical axis refers to KEGG pathway terms. Node color: *P* value.

**Figure 3:**
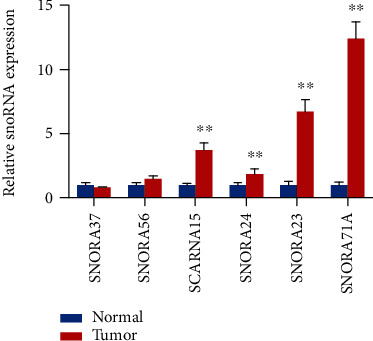
Validation of six candidate snoRNA expression. The relative expression levels of the six candidate snoRNAs were measured by qPCR. The values are expressed as the mean ± SD (*n* = 3). ^∗^*P* < 0.05, ^∗∗^*P* < 0.01.

To better understand the potential function and mechanism of DE snoRNAs, we performed GO and KEGG pathway analysis. The TOP20 GO terms presented some key biological processes including “detection of chemical stimulus involved in sensory perception of smell” and “sensory perception of smell” and “response to stimulus” (Figure 2(a)). Moreover, the KEGG pathway analysis revealed the target genes of the DE snoRNAs were involved in “olfactory transduction,” “glycosphingolipid biosynthesis - ganglio series,” “glycosphingolipid biosynthesis - lacto and neolacto series,” “ribosome,” and “viral myocarditis and tight junction” (Figure 2(b)). To screen the key snoRNAs related to CRC, the six candidate snoRNAs (SNORA37, SNORA56, SCARNA15, SNORA24, SNORA23, and SNORA71A) with high fold change and high abundance were selected for qPCR validation. Only the expression of SCARNA15, SNORA24, SNORA23, and SNORA71A was found to be statistically significant in CRC tissue samples compared to normal tissue samples, and the fold change value of SNORA71A was the highest (Figure 3). Moreover, SNORA71A was located at 20q11.23, and the gene copy number amplifications at this location were found in many cancers such as breast cancer [19], pancreatic adenocarcinoma [20], myeloid malignancies [21], and colorectal cancers [22]. Therefore, we selected the SNORA71A to explore the function role in CRC.

### 3.3. SNORA71A Was Upregulated in CRC Tissues and Cells

**Figure 4:**
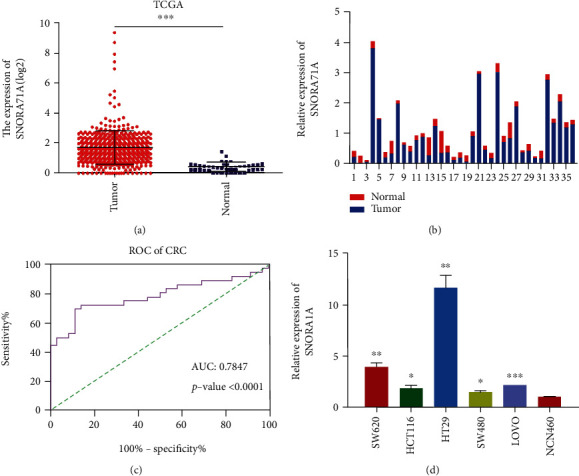
SNORA71A expression was upregulated in CRC tissues and cells. (a) SNORA71A expression between CRC (*n* = 443) and normal colon tissues (*n* = 40) in TCGA. (b) The relative expression levels of SNORA71A were identified by QPCR between 36 pairs of CRC tissues and normal colon tissues. The *x*-axis shows each paired sample. The red represents the normal colon tissues and the blue shows CRC tissues. (c) The diagnosis ability analysis of SONRA71A for CRC via analyzing receiver operating characteristic (ROC) curve. (d) The expression of SNORA71A in human colorectal cancer cells (SW620, SW480, HCT116, LOVO, and HT-29) and normal colonic epithelial cells (NCN460) was measured by qPCR. Data were expressed as the mean ± SD (*n* = 3). ^∗^*P* < 0.05, ^∗∗^*P* < 0.01, and ^∗∗∗^*P* < 0.001.

To further identify whether SNORA71A was upregulated in CRC tissues and CRC cells and was in accordance with the sequencing result and RT-qPCR result, we analyzed data from TCGA, which included 443 CRC cancer tissues and 40 normal tissues. Bioinformatics analysis indicated that the expression level of SNORA71A in CRC tissues was significantly higher than that in normal colon tissues (Figure 4(a)). Subsequently, qPCR assays were utilized to identify SNORA71A expression in 36 CRC tissues and normal tissues. The results showed that the expression level of SNORA71A was also remarkably upregulated in colon cancer tissues compared to normal colon tissues (Figure 4(b)). Next, we analyzed the relationship between SNORA71A expression and clinical parameters of CRC. Statistical analysis suggested that there was a strong association between expression levels of the SNORA71A and TNM stage (*P* < 0.05) and lymph node metastasis (*P* < 0.05), but not with gender, age, and differentiation (Table 3). We also analyzed the diagnosis ability of SNORA71A for CRC via analyzing the receiver operating characteristic (ROC) curve. The tissue expression of SNORA71A might serve as biomarkers for CRC, with the area under curve (AUC) of 0.78 and *P* value less than 0.0001 (Figure 4(c)). At the cutoff value of 5.2, the sensitivity and specificity of SNORA71A diagnosis for CRC were 72.22 and 86.11%, respectively. In addition, the five human CRC cell lines (SW620, SW480, HCT116, LOVO, and HT-29) and NCN460 normal colonic epithelial cells were selected to detect the relative expression of SNORA71A using QPCR. Compared with the NCN460 cells, the expression level of SNORA71A was highest in HT-29 cells, followed by SW620, LOVO, HCT116, and SW480 (Figure 4(d)).

### 3.4. SNORA71A Promoted CRC Cell Proliferation

**Figure 5:**
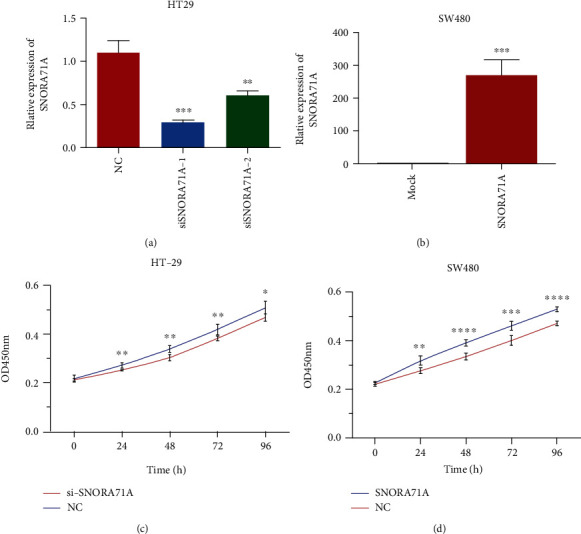
SNORA71A facilitated colorectal cancer cell proliferation. (a) The HT-29 cells were transfected with siSNORA71A-1/2 or NC, and knockdown efficiency of siSNORA71A was evaluated by qPCR assays. (b) After SW480 cells were transfected with SNORA71A expression plasmid or mock plasmid, SNORA71A expression levels were measured by qPCR assays. CCK8 assays were applied to determine HT-29 (c) or SW480 (d) cell proliferation in vitro. Data from at least three independent experiments are shown as the mean ± SD. ^∗^*P* < 0.05, ^∗∗^*P* < 0.01, ^∗∗∗^*P* < 0.001, and ^∗∗∗∗^*P* < 0.0001.

To elucidate the function of SNORA71A in CRC, the effect of SNORA71A on the proliferation ability of CRC cells was investigated. The expression level of SNORA71A was highest in HT-29 cells and lowest in SW480 cells compared with the NCN460 cells (Figures 5(a) and 5(b)). HT-29 cells and SW480 cells were transfected with siSNORA71A and SNORA71A expression plasmid, respectively. QPCR results showed that siSNORA71A and SNORA71A expression plasmid significantly inhibited and increased SNORA71A expression in HT-29 cells and SW480 cells, respectively. The effect of SNORA71A on proliferation ability was further determined by CCK8 assay. SNORA71A knockdown in HT-29 cells decreased the proliferation of CRC cells, and SNORA71A overexpression in SW480 cells promoted proliferation (Figures 5(c) and 5(d)).

### 3.5. SNORA71A Promoted CRC Cell Migration and Invasion

**Figure 6:**
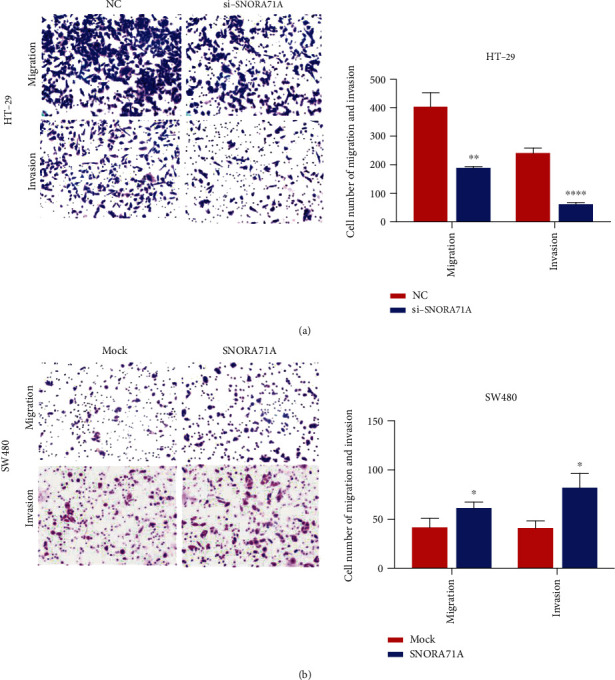
SNORA71A facilitated colorectal cancer cell migration and invasion. (a) Migration and invasion assays were applied to measure HT-29 cell migration and invasion after transfection with siSNORA71A-1/2 or NC, respectively. (b) Migration and invasion abilities of SW480 cells after transfection with SNORA71A expression or mock plasmid were evaluated by migration and invasion assays. Data were presented as the mean ± SD (*n* = 3). ^∗^*P* < 0.05, ^∗∗^*P* < 0.01, and ^∗∗∗∗^*P* < 0.0001.

We further evaluated the effect of the SNORA71A gain- and loss-of-expression on CRC cell migration and invasion. The results indicated that SNORA71A knockdown in HT-29 cells led to significant inhibition of cell migration and invasion ability, and SNORA71A overexpression in SW480 cells promoted migration and invasion ability (Figures 6(a) and 6(b)).

### 3.6. SNORA71A/mRNA/Pathway Interaction Network Analysis

**Figure 7:**
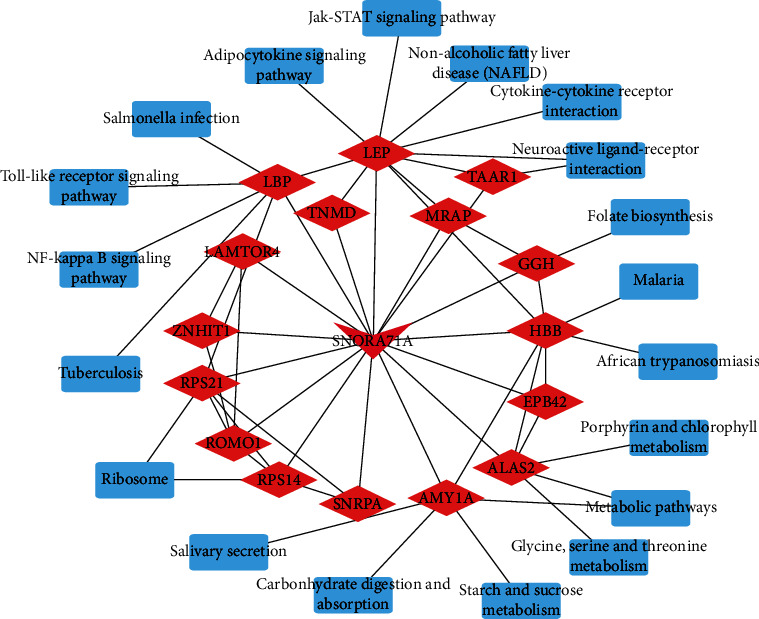
The SNORA71A/mRNA/pathway interaction network constructed and visualized by Cytoscape software. The integrated SNORA71A/mRNA/pathway analysis. The red triangle shows SNORA71A, and the red Rhomboid quadrilateral presents the target mRNAs, respectively. Blue rectangle: signaling pathway.

To further investigate the potential mechanism of SNORA71A, we constructed the SNORA71A/mRNA/pathway interaction network based on the sequencing data. In this complex network, SNORA71A targeted LBP participated in NF-kappa B signaling pathway and Toll-like receptor signaling pathway. SNORA71A targeted LEP participated in Jak-STAT signaling pathway, nonalcoholic fatty liver disease (NAFLD), and cytokine-cytokine receptor interaction. SNORA71A targeted AMY1A participated in metabolic pathways, salivary secretion, carbohydrate digestion, and absorption (Figure 7).

## 4. Discussion

Recent next-generation sequencing and microarray efforts have identified a massive body of noncoding RNAs (ncRNAs), including snoRNAs, which are aberrantly expressed in CRC and play crucial roles in CRC initiation, growth, and metastasis [23–25]. SNORA71A was encoded from the third intron of snoRNA host gene 17 (SNHG17) and was first cloned by Ganot et al. and modulated the pseudouridine of U406 in 18S rRNA [26, 27]. A recent report showed SNORA71A was abnormally expression in NSCLC tissues and functions as an oncogene in NSCLC to facilitate lung cancer cell proliferation, migration, and invasion via MAPK/ERK Pathway [28, 29]. In our study, a total of 45 DE snoRNAs were significantly upregulated, and 62 were significantly downregulated in CRC tissues compared with normal tissues. Among these DE snoRNAs, the selected SNORA71A was remarkably upregulated in CRC tissues and CRC cells via TCGA data analysis and RT-qPCR verification, which was in accordance with the small sequencing result. Additionally, SNORA71A with 72.22% sensitivity and 86.11% specificity might serve as biomarkers for CRC. The SNORA71A had a significant association between expression levels of the TNM stage and lymph node metastasis. The above results suggested that the abnormal expression of SNORA71A might serve as oncogenic factors.

Presently, the role of snoRNAs in the mechanism in CRC remained unknown. The early reports show related pathways of CRC may play key roles in the development of tumorigenesis. For instance, GAS5-derived snoRNAs have a link with p53 levels in colorectal tissue, which indicates that GAS5-derived snoRNAs play an important role in p53-associated signaling pathways in colorectal cancer (CRC) [30]. The expression levels of small nucleolar RNA host gene 15 (SNHG15) may be involved in the NF-*κ*B signaling pathway, induce the epithelial-mesenchymal transition process, and promote renal cell carcinoma invasion and migration [31]. The small nucleolar RNA host gene 1 (SNHG1)/miR-140/TLR4/NF-*κ*B signaling axis plays a crucial role in cholangiocarcinoma tumorigenesis and progression [32]. Here, SNORA71A targeted LBP participated in NF-kappa B signaling pathway and Toll-like receptor signaling pathway. SNORA71A targeted LEP participated in the Jak-STAT signaling pathway. These results imply that SNORA71A may participate in the occurrence and development of CRC.

The intron encoded snoRNAs, in most cases, their expression levels are determined by transcription of host genes [33]. Therefore, the transcription between snoRNA and their host genes might be synchronous. The SNORA71A is located in the third intron of SNHG17; thus, the transcription of SNORA71A might be synchronized to SNHG17. According to the report, SNHG17 is upregulated in CRC tissues and leads to enhancement of the CRC cell proliferation through knocking down P57, suggesting expression of SNORA71A is upregulated in CRC ([34]), whereas the recent study shows the transcription of SNORA71A in NSCLC tissues is independent from its host gene SNHG17 [29]. The link between transcriptions of SNORA71A and SNHG17 needs to be further investigated in CRC

In investigating a connection between snoRNAs and tumorigenesis, the reports indicate snoRNAs are aberrantly expressed and play important roles in CRC. For instance, SNORA42 overexpression results in enhancment of cell proliferation, migration, invasion, anoikis resistance, and tumorigenicity in CRC cells [15]. SNORA21 expression inhibition mediated by CRISPR/Cas9 inhibits cell proliferation and invasion in CRC cells [35]. However, the effect of snoRNAs on initiation and progress in CRC. Here, we further determined in vitro functional significance of SNORA71A in CRC cell lines remains unknown. SNORA71A is required for efficient proliferation, migration, and invasion of CRC cells. Our results indicated that SNORA71A knockdown cells led to a significant inhibition of cell proliferation, migration, and invasion in HT-29. Moreover, SNORA71A overexpression facilitated cell proliferation, migration, and invasion ability in SW480 cells. All these findings further suggested that SNORA71A might serve as an oncogene in CRC.

In conclusion, this study presented differentially expressed profile of snoRNAs in CRC via small RNA sequencing. The SNORA71A was significantly upregulated in CRC tissues and cells. SNORA71A acted as an oncogene and promoted proliferation, migration, and invasion ability of CRC cells.

## Figures and Tables

**Table 1:** Primer sequences and siRNA sequences used in this study.

Gene	Sequences or target sequence (5′-3′)
GAPDH-F	AGAAGGCTGGGGCTCATT
GAPDH-R	TGCTAAGCAGTTGGTGGTG
SNORA71A-F	TGAAAGAGGTTGTCCCCGTG
SNORA71A-R	AAGCTTCAGGGTTCGGATGG
SNORA37-F	TCACTTTGACCAGATGTCTACTGA
SNORA37-R	CCTCAGACGCAGGCTTTCTT
SNORA56-F	TCTAGTCTGGCTCGTGGGAC
SNORA56-R	ACTGTTGCAGACTGACTCCC
SCARNA15-F	GCATGGCCGAATACTGTGTTTTTA
SCARNA15-R	AAGGGAAGACTGCTTTTGCAT
SNORA24-F	CTTTGGGACCTGTCAGCCG
SNORA24-R	CTAGGAAGGGAGACTGCCAC
SNORA23-F	ACATCATGCGGCCAAAGAGT
SNORA23-R	TGTGGGAATTTGGAGGCTGG
si-SNORA71A-1	CCUGCAUCCGAAAGUGAUCTT (sense)
si-SNORA71A-1	GAUCACUUUCGGAUGCAGGTT (antisense)
si-SNORA71A-2	GCCUAGGUCAUUGAUAGUGTT (sense)
si-SNORA71A-2	CACUAUCAAUGACCUAGGCTT (antisense)
SNORA71A-NC	UUCUCCGAACGUGUCACGUTT (sense)
SNORA71A-NC	ACGUGACACGUUCGGAGAATT (antisense)

**Table 2:** Summary of snoRNA sequencing datasets.

Sample name	Total reads	Clean reads	GC (%)	Mapped reads (reference genome)	Mapped reads (Rfam database)
N1	133,679,270	43,525,148 (33%)	47	5,901,044 (97%)	10,099,878 (88%)
N2	156,613,612	44,614,556 (28%)	48	8,544,838 (97%)	11,520,278 (88%)
N3	30,570,028	25,687,700 (84%)	48	8,134,694 (97%)	10,916,943 (91%)
T1	48,668,926	39,956,661 (82%)	48	7,474,852 (98%)	10,713,059 (85%)
T2	44,924,294	37,162,849 (83%)	48	9,330,200 (97%)	12,687,170 (92%)
T3	25,934,552	19,599,274 (76%)	49	10,416,571 (97%)	12,936,944 (91%)

**Table 3:** The relationship of SNORA71A expression and clinical parameters in colorectal cancer.

Clinicopathological parameter	Feature	Total	SNORA71A low	SNORA71A high	*P* values
All		36	18	18	
Gender	Male	18	8	10	0.111111
	Female	18	10	8	0.738883
Age	<60 years	14	6	8	0.116883
	>60 years	22	12	10	0.732440
Differentiation	Poor+moderate	30	15	15	0.200000
	Well	6	3	3	0.654721
TNM stage	I/II	18	13	5	5.444444
	III/IV	18	5	13	0.019631^∗^
Lymph node metastasis	No	20	14	6	5.512500
	Yes	16	4	12	0.018881^∗^

The chi-square test was used for analysis. The average expression level served as the cut-off point. ^∗^The results were statistically significant (*P* < 0.05).

## Data Availability

The datasets used and/or analyzed during the current study are available from the corresponding author on reasonable request.

## Supplementary Materials

Supplementary Materials The 107 differentially expressed snoRNAs in colorectal cancer tissues compared to normal tissues.
